# Identifying health policy and systems research priorities on multisectoral collaboration for health in low-income and middle-income countries

**DOI:** 10.1136/bmjgh-2018-000970

**Published:** 2018-10-10

**Authors:** Douglas Glandon, Ankita Meghani, Nasreen Jessani, Mary Qiu, Sara Bennett

**Affiliations:** 1 Department of International Health, Johns Hopkins Bloomberg School of Public Health, Baltimore, Maryland, USA; 2 Department of Health, Behavior and Society, Johns Hopkins Bloomberg School of Public Health, Baltimore, Maryland, USA

**Keywords:** research priorities, priority setting, health policy, health systems, developing countries

## Abstract

**Introduction:**

While efforts to achieve Universal Health Coverage (UHC) and the Sustainable Development Goals (SDGs) have reinvigorated interest in multisectoral collaborations (MSCs) among the global health and development community, there remains a plethora of questions about how best to conceptualise, plan, implement, evaluate and sustain MSCs. The objective of this paper is to present research priorities on MSC for health from researchers and policymakers around the globe, with an emphasis on low-income and middle-income countries.

**Methods:**

The authors identified 30 priority research questions from two sources: (1) 38 review articles on MSC for health, and (2) interviews and focus groups with a total of 81 policymakers, including government officials (largely from ministries of health and state/provincial departments of health, but also offices of planning, public service, social development, the prime minister and others), large multilateral or bilateral organisations, and non-governmental organisations. In a third phase, questions were refined and ranked by a diverse group of researchers from around the globe using an online voting platform.

**Results:**

The top-ranked questions focused predominantly on pragmatic questions, such as how best to structure, implement and sustain MSCs, as well as how to build stakeholder capacity and community partnerships. Despite substantial variation between review articles, policymakers’ reflections and online ranking by researchers, two topics emerged as research priorities for all three: (1) leadership, partnership and governance structures for MSCs; and (2) MSC implementation strategies and mechanisms. The review articles underscored the need for more guidance on appropriate study designs and methods for investigating MSCs, which may be a prerequisite for other identified research priorities.

**Conclusion:**

These findings could inform efforts within and beyond the health sector to better align research objectives and funding with the evidence needs of policymakers grappling with questions about how best to leverage MSCs to achieve UHC and the SDGs.

**Key questions**

**What is already known?**

- Despite the widespread interest in multisectoral collaborations (MSCs) to achieve Universal Health Coverage and the Sustainable Development Goals, there remains a substantial knowledge gap about how best to plan, implement and evaluate them.

**What are the new findings?**

- This paper outlines a prioritised list of proposed research questions on MSCs based on a literature review and input from policymakers and researchers around the world.

**What do the new findings imply?**

- The highly pragmatic focus of many of the priority questions underscores the need for actionable, evidence-based guidance on MSCs for policymakers and practitioners.
- At the same time, the noted gap in research methods for studying MSCs suggests that some foundational methodological work may be needed before some of the other priority questions can be answered.

## Introduction

While researchers and practitioners of the various historical antecedents to today’s ‘global health’ have written about the importance of considering multisectoral determinants of health for over a hundred years, the level of global interest in the topic has waxed and waned over time.^1^ This is due, at least in part, to a combination of technological advances, social movements and other factors related to national and international politics.^1^ The concept of ‘intersectoral action for health’ was formally introduced at the International Conference on Primary Health Care in Alma Ata, Kazakhstan, in 1978,^2^ and has since been incorporated into many countries’ official policy frameworks and highlighted in a variety of international conferences and initiatives.^2–4^

Renewed interest in multisectoral collaboration (MSC) in recent years has coincided with ongoing efforts to achieve Universal Health Coverage (UHC)^5^ as well as the launch of the Sustainable Development Goals (SDGs) framework,^6^ which underscores the interdependence of multiple sectors in achieving many of the agreed objectives and targets.^7–9^ In addition to the explicit focus on health in SDG 3 ‘Good health and well-being’, many of the other SDGs indicate critical linkages to health; examples include the interrelationship between poverty and health (SDG 1), addressing malnutrition as a part of reducing hunger (SDG 2), the role of health in early childhood development and education (SDG 4), achieving gender equality in access to healthcare (SDG 5) and so forth. Even within SDG 3, there are multiple targets that will necessitate MSCs for health. For instance, reducing the number of deaths and injuries due to road traffic accidents will likely require collaboration with departments of transport and urban planners; preventing and treating substance abuse will likely require collaboration with law enforcement agencies; and so forth.

Building on these observations, the term ‘sector’ in this paper refers to the primary topical or thematic domain of an institution or stakeholder group (eg, health, education, agriculture, transportation and so on), as opposed to its organisational type or legal status (eg, government, business, non-profit, civil society organisation). Thus, for the purpose of this paper, we treat MSC as synonymous with the consensus definition of intersectoral action for health from the 1997 international conference ‘Intersectoral Action for Health: A Cornerstone for Health-for-All in the Twenty-First Century’:

a recognized relationship between part or parts of the health sector with part or parts of another sector which has been formed to take action on an issue to achieve health outcomes (or intermediate health outcomes) in a way that is more effective, efficient, or sustainable than could be achieved by the health sector acting alone. (National Centre for Health Promotion 1995, cited in ref 3)

The authors’ aim in focusing on collaborating across topical domains is not to diminish the significance of partners’ organisational types in shaping a potential collaboration. On the contrary, the definition allows for any combination of organisational types among partner institutions, including, for example, public–private partnerships involving government entities and for-profit businesses, public–non-profit partnerships, and whole-of-government initiatives. Given that there is already an extensive body of literature on these various types of multistakeholder partnerships, the emphasis on topical domains is intended to highlight an additional layer of complexity that has been widely recognised as important but that has received much less attention from researchers to date.

Anticipating the important and ongoing role of the SDGs in shaping government priorities and policies, the Alliance for Health Policy and Systems Research (AHPSR) commissioned work to examine research priorities linked to the SDGs. This work builds on substantial prior research priority-setting work by the AHPSR that includes the identification of priorities for health financing,^10^ human resources for health^11^ and access to medicines.^12^ Given the breadth and complexity of the SDGs, this current round of health systems research priority-setting focused on three relatively novel themes that are reflected—either explicitly or implicitly—across multiple SDGs but that have not, to the authors’ knowledge, been the focus of previous priority-setting efforts. These themes, which were discussed and agreed between the research team and the AHPSR, include social protection for health (Qiu *et al*, forthcoming), social accountability (Scott *et al*, forthcoming) and MSC. This paper reports on research priorities linked to the third theme, MSC.

In the authors’ view, the high level of global interest in MSCs, combined with a substantial gap of evidence about how best to plan, implement and evaluate them, calls for a targeted effort to advance this body of knowledge. Towards that end, this study outlines key health policy and systems research (HPSR) priorities to aid low-income and middle-income countries (LMICs), global partners and research funders in leveraging MSC for health as part of an overall strategy and learning agenda in pursuit of UHC and the SDGs.

## Methods

Based on the assumption that HPSR is most meaningful when it responds to real policy needs and most impactful when it engages key decision-makers from the start, we sought out policymaker perspectives on research priorities to complement insights from the existing literature. Our methods drew on previous overviews of reviews,^10 11 13^ policymaker consultations^10 11^ and priority-setting processes,^10 11 14^ with the notable adaptation of the latter by using an online platform for soliciting input from the global research community in order to encourage a more geographically inclusive and efficient process. Given time and resource constraints, combined with the fact this global exercise is intended as a reference for refinement at the country level, we chose not to engage a wider range of stakeholders^15 16^ or pursue a more deliberative^17 18^ approach to priority-setting, although we recommend that such approaches be considered for future region-specific or country-specific exercises.

The study was carried out in three main phases: phase I (overview of reviews), in which we conducted an overview of existing reviews on MSC for health to identify proposed areas for future research; phase II (consultations with policymakers), in which we interviewed policymakers to seek their views on evidence needs related to MSC for health; and phase III (identification, refinement and ranking of research questions), in which we synthesised findings from the two first phases in order to develop a list of potential research questions which were then refined and prioritised by a diverse range of researchers with relevant experience from around the world.

### Phase I: overview of reviews

#### Search strategy

Databases searched included PubMed, Embase, Scopus, PAIS International, Social Science Abstracts, PsycINFO, WHO Global Health Regional Indexes and Ovid’s Global Health database. The search strategy involved selecting articles in which the title or abstract matched a combination of controlled vocabulary and keyword terms such that at least one term was matched for all of the following concepts (each of which contained a subset of relevant terms, which are listed in online supplementary appendix 1; further discussion of the choice of search terms is included in online supplementary appendix 2):

10.1136/bmjgh-2018-000970.supp1 Supplementary data

10.1136/bmjgh-2018-000970.supp2 Supplementary data

1. Intersectoral/multisectoral.
2. Health or nutrition.
3. Government/public.
4. Review/synthesis.

The search results were limited to studies published between January 2000 and March 2017. Records from all databases were imported on 16 March 2017. All duplicates were removed and unique citations were exported to Microsoft Excel for screening.

#### Study selection and criteria

The inclusion criteria for the overview of reviews were as follows: (1) published in English, Spanish or Portuguese, with an abstract available in English; (2) published in the peer-reviewed or grey literature; (3) described collaborations that include institutions within the health sector plus one or more non-health sectors; (4) described collaborations that include at least one key objective or outcome that relates to human health, well-being or a determinant of health; and (5) described collaborations that identify at least one official government office/department/entity of the country in which action is being undertaken as a key actor/stakeholder. Articles were excluded if considered not to be a review (including, for instance, commentaries, case studies, project or policy narratives, and articles with no described methodology for a review). There were no restrictions related to the income level of the countries in which the underlying research was based.

**Figure 1:**
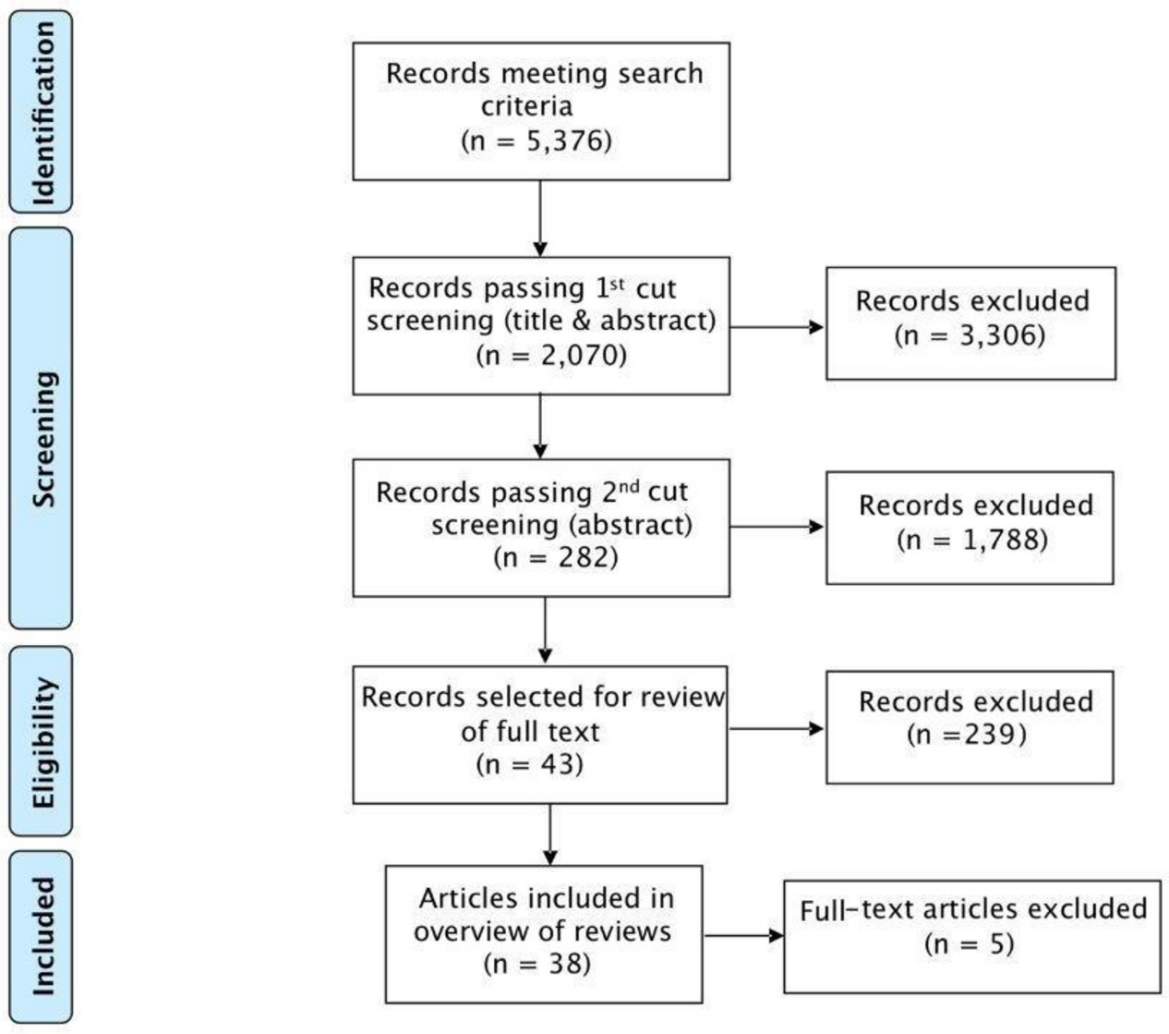
Diagram of search results.

This process of record identification, screening and eligibility assessment is outlined in figure 1. In the screening step, DG and AM reviewed record titles and abstracts to remove any articles that obviously did not meet the inclusion criteria, including a rapid ‘first cut’ followed by a slower and more involved ‘second cut’. In the eligibility step, DG, AM and SB each reviewed the abstracts of the 282 remaining articles and independently voted on the eligibility of each for full-text review. Discrepancies were discussed in order to produce a consensus decision on each article. An additional five articles were excluded after full-text review because they focused only indirectly or peripherally on MSC.

#### Data extraction

Key information from articles (eg, metadata, study countries/region, study question(s), purpose of MSC and so on) was extracted using a Microsoft Office Excel-based template. The full set of data fields extracted are listed in online supplementary appendix 3. We considered and recorded the quality of each review during the data extraction process; however, reviews were not excluded for being of poor quality. For each field, relevant article text was copied and pasted verbatim into the template.

10.1136/bmjgh-2018-000970.supp3 Supplementary data

#### Identifying specific research questions from reviews

From the 38 articles reviewed, 110 specific research topics or questions related to MSC were identified from the relevant segments of extracted article text, typically from the results and/or discussion sections. In instances where these topics were expressed in the form of issues requiring further investigation, the study team paraphrased them as questions. Similar questions were grouped, first by overarching theme, and then, within those themes, by research need (see online supplementary appendix 4). Questions deemed substantially similar within the same thematic and research need group were then consolidated to avoid redundancy in the prioritisation exercise. As part of this step, questions that focused on a particular health issue or country were framed more broadly to increase their applicability across topical and geographical contexts. Although this process resulted in question phrasing that differed slightly from the original sources, the research team strived to preserve the same meaning to the extent possible.

10.1136/bmjgh-2018-000970.supp4 Supplementary data

### Phase II: consultations with policymakers

We invited senior-level policymakers (typically directors and deputy directors, but including some secretaries, assistant secretaries and special advisors) from around the world, as well as senior staff from large multilateral or bilateral organisations and non-governmental organisations, to participate in the process. Target respondents were identified in several ways: (1) participant lists at two major global conferences attended by the study team: Health Systems Global 2016 (Vancouver, Canada) and the Prince Mahidol Awards Conference 2017 (Bangkok, Thailand); (2) recommendations from colleagues at the AHPSR; and (3) through relationships with colleagues based in India, South Africa, Lebanon and Argentina. While there was no targeted number of respondents, we sought to include balanced geographical representation across LMICs and thus continued inviting participants until we felt that we had achieved a relatively equitable distribution. The majority of participants were from the health sector, although some of the public sector officials came from other ministries, such as planning, environmental affairs, and public service and administration.

Participants were contacted via email, by phone or in person. Indepth interviews (IDIs) were conducted face to face where possible, and by phone in instances where geographical location posed a challenge. Focus group discussions (FGDs) were held in lieu of IDIs in Bahrain and Jordan in order to enable the participation of a greater number of policymakers.

IDI guides included questions on context-relevant health systems challenges anticipated with respect to meeting the SDGs, policy changes being considered to mitigate these and specific questions around three themes, of which MSC was one. The results related to the other themes are published elsewhere. Within the MSC theme, specific questions of interest were around challenges, policy considerations, and information and evidence needs. IDIs and FGDs were audio-recorded on participants’ permission. Audio files were coded with a study ID and then used to support the preparation of detailed notes, including relevant segments of transcribed text, related to the study topic. This combination of detailed notes and direct quotes was used to populate a matrix of results, with rows representing policymakers and columns representing content areas, allowing the team to use a framework analysis approach^19^ to identify policymaker research priorities. All de-identified audio files and transcripts were stored in a secured, limited access, password-protected electronic database.

### Phase III: identification, refinement and ranking of research questions

#### Identification

In the third phase, the study team incorporated the identified evidence needs and questions from the policymakers into the Excel spreadsheet of the questions from the review articles. In situations where the policymaker questions did not fit neatly within one of the existing ‘research need’ groupings, a new one was created. The study team completed this process over four iterative rounds of discussion and refinement, including changes to the grouping of the research questions as well as the wording of the identified research needs. The process resulted in the identification of 13 global themes, 21 research needs and 30 research questions, as illustrated in online supplementary appendix 4.

#### Refinement and ranking

We then invited a diverse group of health systems researchers from around the globe to refine and rank the research questions identified in phases I and II using an online collaboration platform called Co-Digital (www.codigital.com), which is designed to facilitate generation, revision and prioritisation of ideas by a large number of participants in a user-friendly format. Participants for this activity were identified through targeted invitations and solicitations on the Health Systems Global web page (www.healthsystemsglobal.org) and on Twitter. The criteria for identifying participants included that they should have health systems research experience and be familiar with MSC issues. We also sought to identify a mixed pool of participants, reflecting diverse regions, disciplines and country economic contexts. Of the 127 people who indicated interest, 72 met the above-mentioned criteria. As part of the invitation to contribute to the prioritisation process, participants were provided with a draft of the overview of reviews, as well as a Microsoft Excel spreadsheet with the collated list of research questions from the overview of reviews and policymaker consultations.

At the first stage, which lasted from 2 October to 8 October 2017, the participants were invited to propose edits to the research questions developed by the study team and then to vote on those edits in real time, which resulted in an iterative refinement of the question framing and wording. Of the 72 eligible respondents, 26 participated and contributed to 79 unique edits, and cast a total of 392 votes for all the edits during this phase. Once the refining stage was complete, the study team reviewed the final iteration of each research question and incorporated all edits that remained consistent with the original intent of the question. In instances where this did not happen, the study team reverted to the original or a previous iteration of the question. The final set of research questions was then uploaded to the Co-Digital platform for the second stage.

At the second stage, which took place from 16–22 October 2017, the same 72 individuals were invited to rank the edited set of questions in order of priority through a series of pairwise comparisons. Consistent with other AHPSR exercises,^10 11^ the study team asked participants to consider several criteria to guide their ranking, including (1) potential benefits or impact of research on a specific question; (2) tractability of the research question; and (3) the extent to which answering the question would benefit the poor and marginalised communities. These criteria were intentionally kept rather general to allow respondents some leeway to apply their own interpretation. They also align with the first two of the three broad categories of health research priority-setting (HRPS) criteria identified by Viergever *et al* 20: public health benefit, feasibility and cost.

Through several rounds of this pairwise ranking process, high priority research questions were determined. A total of 30 individuals participated in the ranking phase, casting a total of 651 votes. The final scores for the ranking were unweighted and calculated based on the number of times a research question won when competing directly with another research question. The final ranking of the research questions was shared with the participants, who were invited to share their reflections on both the results as well as the process.

## Results

### Phase I: overview of reviews

#### Results of search strategy

Of the 38 articles reviewed, 21 exclusively or primarily focused on high-income countries (HICs), as highlighted in table 1. Another 11 articles had a non-specific, global focus or included countries across a mixture of income categories. Only six of the reviews included an explicit focus on LMICs, including one that focused on MSC policies in a single upper-middle-income country (Brazil).^21^ Of the 38 articles, 24 were literature reviews, which is somewhat loosely defined here, as many of the authors only vaguely described their review methods and some supplemented findings derived from the literature with additional data sources, such as stakeholder interviews.

**Table 1:** Overview of reviews

Review (author, year)	Type of review (as identified by the authors)	Country focus	Domain/Theme
Martin-Moreno *et* *al*,^32^ 2016	Literature review	Global	Cross-cutting
Ng and Ruger,^33^ 2011	Literature review	Global	Cross-cutting
Woulfe *et* *al*,^34^ 2010	Literature review	Global/HIC	Cross-cutting
Casanovas *et* *al*,^35^ 2013	Narrative review	Global	Early childhood development
Buse and Hawkes,^36^ 2015	Literature review	Global	Non-communicable diseases
Williams,^37^ 2009	Literature review	HIC	Criminal justice
Berends *et* *al*,^38^ 2016	Literature review	HIC	Cross-cutting
Carey *et* *al*,^39^ 2014	Meta-synthesis	HIC	Cross-cutting
Hayes *et* *al*,^40^ 2012	Meta-analysis	HIC	Cross-cutting
Hendriks *et* *al*,^41^ 2014	Literature review	HIC	Cross-cutting
Kindig *et* *al*,^42^ 2003	Literature review	HIC	Cross-cutting
Lorenc *et* *al*,^43^ 2014	Systematic review	HIC	Cross-cutting
van Herten *et* *al*,^44^ 2001	Literature review	HIC	Cross-cutting
de Leeuw,^45^ 2017	Literature review	HIC	Cross-cutting/HiAP
Gase *et* *al*,^46^ 2013	Literature review	HIC	Cross-cutting/HiAP
Gase *et* *al*,^47^ 2017	Literature review	HIC	Cross-cutting/HiAP
Gannon-Leary *et* al,^48^ 2006	Literature review	HIC	Health and social services
Mackie and Darvill,^49^ 2016	Systematic review	HIC	Health and social services
Green *et* *al*,^50^ 2014	Literature review	HIC	Injury and disability
Whiteford *et* *a* *l*,^51^ 2014	Systematic review	HIC	Mental Health
Bergeron and Lévesque,^52^ 2012	Literature review	HIC	Physical activity
Dawson *et* *al*,^53^ 2015	Literature review	HIC	Physical activity
Ndumbe-Eyoh and Moffatt,^54^ 2013	Literature review	HIC	Social determinants
de Azevedo *et* *al*,^21^ 2012	Non-systematic review	LMIC	Health promotion
Bowen *et* *al*,^55^ 2013	Literature review	LMIC	Climate change
Du *et* *al*,^56^ 2015	Literature review	LMIC	Food security/nutrition
Hurley *et* *al*,^57^ 2016	Literature review	LMIC	Food security/nutrition
Phuka *et* *al*,^58^ 2014	Literature review	LMIC	Food security/nutrition
Hongoro *et* *al*,^59^ 2008	Literature review	LMIC	HIV/AIDS
Corbin *et* *al*,^60^ 2016	Scoping review	Mixed	Cross-cutting
Magee,^61^ 2003	Literature review	Mixed	Cross-cutting
Rantala *et* *al*,^62^ 2014	Scoping review	Mixed	Cross-cutting
Ehrenberg and Ault,^63^ 2005	Literature review	Mixed	Neglected diseases
Pedrana *et* *al*,^64^ 2016	Scoping review	Mixed	Social determinants
Shankardass *et* *al*,^65^ 2012	Scoping review	Mixed	Social determinants
Chircop *et* *al*,^66^ 2015	Scoping review	HIC	Review of frameworks
Klassen *et* *al*,^67^ 2010	Systematic review	HIC	Review of frameworks
Cohen and Marshall,^68^ 2017	Scoping review	Mixed	Review of frameworks

HIC, high-income country;HiAP, Health in All Policies;LMIC, low-income and middle-income country.

The articles included a combination of those that were explicitly focused on the structure, process and mechanisms of MSC (labelled in table 1 as ‘cross-cutting’) as well as those that focused on MSC associated with a specific health topic or policy area. The most common theme was ‘cross-cutting’ (16 articles), which included 3 articles specifically focusing on Health in All Policies, followed by social determinants of health (3 articles), food security and nutrition (3 articles), health and social services (3 articles), and physical activity (2 articles).

There was also one article focusing on MSC in each of the following areas: early childhood development, non-communicable diseases, criminal justice, injury and disability, mental health, health promotion, climate change, HIV/AIDS, and neglected diseases. Three articles reviewed existing conceptual frameworks to inform the MSC literature.

#### Proposed research questions from reviews

Of the 30 research questions, those most commonly suggested in the review articles related to study designs and methods, governance structures and processes, contextual factors, strategies and mechanisms for implementing MSC, and measuring the additional impact of MSC beyond single-sector interventions (see table 2). Notably, the single most frequently suggested research question was about how best to conduct research on MSCs, indicating the need for further methodological work which may, at least to some extent, be a prerequisite for answering some of the other questions.

**Table 2:** 10 most commonly identified research questions from review articles

#	Research question	Suggested in (n) articles
1	Which study designs and methods are best suited to understanding multisectoral collaborations, their governance, functioning and outcomes?	18
2	What types of leadership, partnership and governance structures and processes are most effective for multisectoral collaboration?	10
2	How do contextual factors such as institutional arrangements, governance arrangements and partnership experiences affect the success (or failure) of multisectoral collaborations?	10
4	Which strategies and mechanisms are effective in supporting the implementation of multisectoral collaborations for health? (eg, enabling legislation, policy mandate, decentralised control, accountability and incentive mechanisms, dedicated resources, training/skill development and so on).	9
5	What is the additional impact of multisectoral collaboration on health and health equity outcomes as compared with single-sector approaches?	8
6	What are the key conditions or drivers for the formation of multisectoral partnerships (eg, political context, motivating factors for partners and so on)?	6
7	How can we best enhance the capacity of stakeholders concerned about multisectoral action for health (such as health advocates or health practitioners) to engage in multisectoral initiatives?	5
7	How can multisectoral collaborations improve health equity and social determinants of health?	5
9	How does the use of evidence differ across different sectors and how can we make health evidence more accessible and actionable in other sectors?	4
9	Which theories and/or conceptual frameworks are most valuable in understanding multisectoral issues?	4

### Phase II: consultations with policymakers

Of the 85 people invited for the IDIs, 54 ultimately participated; of the 33 people invited to one of the two FGDs, 27 participated. Non-participation was due to a combination of scheduling challenges and non-response to invitations. The geographical distribution of actual participants spanned five WHO regions and was largely similar to those invited. In some of the IDIs, the respondents did not cover all three of the focal themes, so the number of respondents that addressed MSC was slightly fewer. Approximately 28% of the interviewed respondents were women, which is comparable with available global estimates of the proportion of women in senior policymaking positions; according to the Inter-Parliamentary Union, 23.8% of parliamentary seats globally are held by women, with substantially lower proportions in many LMICs and substantially higher proportions in the Nordic countries.^22^ Further respondent details can be found in online supplementary appendix 5.

10.1136/bmjgh-2018-000970.supp5 Supplementary data

#### Policymaker identified research areas

Policymakers were keen to explore ideas about how to convince other sectors of the relevance of health to them. For instance, the respondent from Pakistan expressed a desire to explore how achieving good indicators in health contributes to improvements in the labour force and other industries. Similarly, a policymaker from Kenya highlighted the cascading, intersectoral consequences of inadequate food security during famines, stating that “if the agricultural sector does not invest enough, the cases end up in the hospital and the burden goes to the health sector to treat malnutrition.” This in turn, he added, can affect education because of the link between malnutrition and low IQ. Several policymakers, including those from Liberia, Zimbabwe and Thailand, noted that learning and collaboration of this type is a notable challenge given the siloed structure of government and the various ways in which ministries and sectors operate. There was a recognition by one respondent (Bahrain) that operationalising MSCs requires a whole-of-government approach in order for questions posed by sector-specific policymakers to be adequately deliberated. Continuing the thought, this respondent asked about the implications of MSC for the sharing of information and human and financial resources between ministries, as well as the appropriate roles of specific cross-cutting institutions, like the civil service bureau or the ministry of finance. Similarly, the Bhutanese respondent indicated that the cost of MSCs would be important to take into consideration, yet there is little or no data on this.

Policymakers noted that research on governance and leadership would be critical to better understand what types of mechanisms would be able to support and promote MSCs. For instance, respondents from South Africa and Myanmar raised queries around transformative leadership and the accompanying roles, responsibilities and decision-making processes that had been effective in facilitating MSC in other countries. Questions around innovative models of governance and structures of government to support effective MSC—in addition to the aforementioned points about flows of financial and human resources—were also raised by the respondents from the Caribbean, Bahrain, Somalia, Indonesia and India and some development partners. Furthermore, concerns around how to build a sense of collective ownership for MSC when activities reside within one ministry for administrative purposes were also raised.

While there have been multiple attempts to develop MSCs, some policymakers felt there were very few documented examples of effective governance structures for MSCs, particularly at the national level (Development Partner). Respondents from South Africa, India and representing one of the Development Partners indicated wanting to learn from countries they believed had already had achievements or significant experience in this field, such as Argentina and Thailand, so that these learnings could be adapted to their local contexts. One respondent, however, felt that the lack of context-specific evidence is currently being used as an excuse for countries not willing to move forward on more MSCs.

Policymakers were curious about how to ensure that MSCs are not just the responsibility of the government but that the approach takes into consideration the roles of multiple stakeholders in ensuring its success. For example, a respondent from Ghana asked about how to involve those outside the national government, such as traditional leaders, district assembly members, and community opinion leaders. Similarly, a respondent from Indonesia stated that there is a need for research on how to develop policies that engage stakeholders beyond the public sector. The role of the private sector and mutual benefit for public–private partnerships appeared numerous times across respondents from a variety of contexts, including Argentina, Kenya and Bhutan. Accompanying this was a recognition about the challenges that MSCs pose in terms of articulating the roles of the various partners and, in parallel, mechanisms of accountability.

Finally, the issue of scale-up emerged as important when talking about the various aspects of MSCs. The respondent from Bhutan mentioned having significant success in MSCs at the local level but less at the national level. This was echoed by a respondent from a Development Partner who noted similar experiences across the Americas. Respondents from Indonesia, Kiribati, Somalia and a Development Partner highlighted the importance of institutionalisation and sustainability of MSCs in the face of limited resources, parallel programme, a siloed culture of work and frequent rotation of government officials.

The five most commonly identified types of research questions (see table 3) from the policymaker interviews and FGDs all related to strategic and operational aspects of planning for and implementing MSCs. For instance, the single most frequently suggested research question related to identifying the types of governance structures and processes that are most conducive or effective for MSCs, followed by questions about the best strategies or mechanisms for implementing MSCs, approaches to increase the commitment of MSC partners, engaging in MSCs with private sector partners, and understanding the appropriate role for the ministry of health in MSCs relative to other government ministries.

**Table 3:** 10 most commonly identified research questions from policymakers

#	Research question	Identified in (n) discussions
1	What types of leadership, partnership and governance structures and processes are most effective for multisectoral collaboration?	13
2	Which strategies and mechanisms are effective in supporting the implementation of multisectoral collaborations for health? (eg, enabling legislation, policy mandate, decentralised control, accountability and incentive mechanisms, dedicated resources, training/skill development and so on).	8
2	In formal multisector partnerships, what can be done to increase the commitment of members through incentives and other means?	8
4	What are the main differences in multisectoral collaborations involving private sector partners versus public sector only?	7
5	What is the appropriate role for the Ministry of Health in multisectoral collaborations vis-à-vis other ministries and how does this vary across topics/contexts?	6
6	How can indicators and information systems be harmonised across partners in a multisectoral collaboration?	5
7	What are the factors that help to sustain multisectoral collaborations over time?	4
8	How can initiators of multisectoral collaboration determine the appropriate scope of the partnership (eg, number of partners to include, level of involvement of each)?	3
8	How does multisectoral collaboration at the local level differ (eg, in terms of challenges, processes) from the national level?	3
8	What is the impact of good health or health services on the ability of other sectors (outside of health) to achieve their Sustainable Development Goals?	3
8	What is the additional impact of multisectoral collaboration on health and health equity outcomes as compared with single-sector approaches?	3

### Phase III: identification, refinement and ranking of research questions

#### Priority research questions identified from reviews and policymaker consultations

The final set of research questions (online supplementary appendix 4) can be described as those examining strategies for the effective implementation of MSCs, the role of different actors/stakeholders in the formation and implementation of MSCs, and the effects of contextual factors on the success and sustainability of MSCs. Questions also focused on how data and evidence could be harmonised and made accessible and actionable across sectors, how sustained engagement of different stakeholders in MSCs could be incentivised, and the impact of MSCs on health outcomes, health equity and the social determinants of health.

#### Final ranking of priority research questions

Table 4 lists the top 10 priority research questions based on participant ranking. Overall, these questions tended to focus on pragmatic ‘how to’ guidance. Case in point, 7 of the 10 questions ask about how best to structure, implement and sustain MSCs, as well as about stakeholder capacity building, the role for community-based partnerships, key implementation challenges and important contextual factors to consider. Two of the top 10 questions are about outcomes, one focusing on how MSCs affect health equity and social determinants of health and the other considering how interventions targeting non-health SDGs affect health outcomes. The two remaining questions have more of a research focus: one asks about how to increase evidence sharing across sectors, while the other asks which research methods are best suited for understanding MSCs. Of note, there was a fairly narrow spread in the final percentage scores for the top 10 ranked questions, with question 1 being selected in nearly 68% of the pairwise comparisons and the two questions tied for the number 10 slot being selected nearly 51% of the time. There was also a fairly gradual drop-off in scores for the remaining questions, with questions 12–20 scoring in the 40%–49.9% range, questions 21–26 scoring in the 30%–39.9% range, and the remaining question scoring below 30% (online supplementary appendix 6).

10.1136/bmjgh-2018-000970.supp6 Supplementary data

**Table 4:** Top 10 ranked research questions on multisectoral collaboration for health

Rank	Research question	Unweighted final score (%)
1	Which strategies and mechanisms are effective in supporting the implementation of multisectoral collaborations for health? (eg, enabling legislation, policy mandate, decentralised control, accountability and incentive mechanisms, dedicated resources, training/skill development and so on).	67.9
2	What factors are necessary for sustaining multisectoral collaborations over time?	63.0
3	How does the use of evidence differ across different sectors and how can we make health evidence more accessible and actionable in other sectors?	62.7
3	What is the role of community-based partnerships and initiatives in driving multisectoral collaborations for health?	62.7
5	What types of leadership, partnership and governance structures and processes are most effective for multisectoral collaboration?	60.0
6	What are the key challenges to implementing multisectoral programme and interventions to address health issues (eg, food security, non-communicable diseases, HIV/AIDS)?	59.7
7	How do contextual factors such as institutional arrangements, governance arrangements, democratic values and partnership experiences affect the success (or failure) of multisectoral collaborations?	53.0
8	How can we best improve the capacity of stakeholders involved in multisectoral action for health (such as health advocates or health practitioners) to engage in and also promote multisectoral initiatives?	52.6
9	Which study designs and methods are best suited to understanding multisectoral collaborations, their governance, functioning and outcomes?	51.8
10	How do multisectoral collaborations affect health equity and social determinants of health?	50.9
10	How do interventions that target non-health Sustainable Development Goals affect health outcomes?	50.9

### Summary of priority questions across all sources

**Figure 2:**
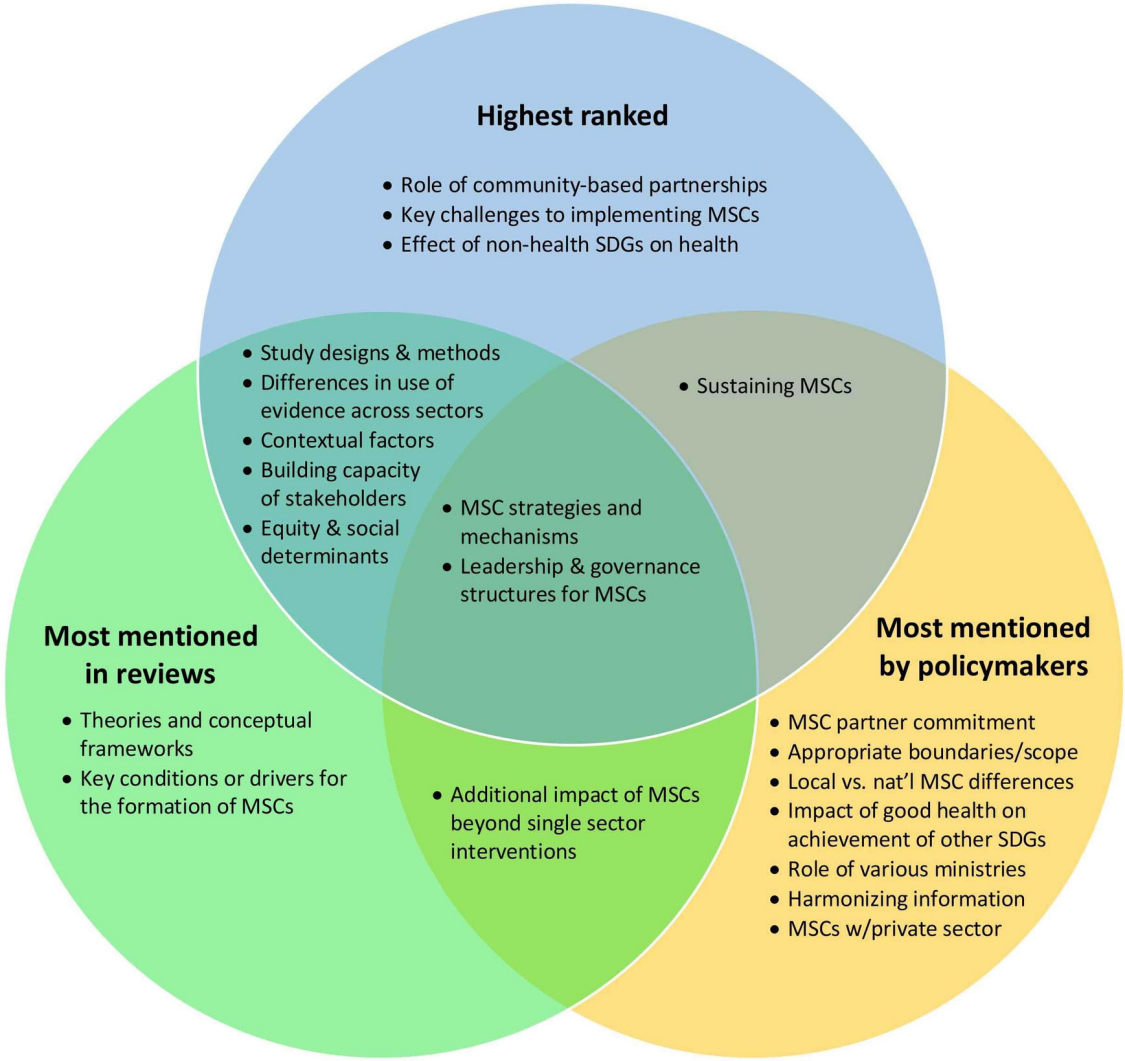
Overlap of research question rankings and frequency of mention. The text in figure is paraphrased from the actual research questions in order to increase the legibility of the diagram. Please refer to tables 2–4 for the full questions. MSCs, multisectoral collaborations; SDGs, Sustainable Development Goals.

Of the initial 30 questions, 27 were identified by more than one source (whether article authors or policymakers), although with variations in framing and/or wording. Some of the questions were mentioned frequently, with 13 out of the 30 identified by 5 or more sources, and 6 out of the 30 identified by 10 or more sources (see online supplementary appendix 7). As a result, it is possible to compare the rankings with the numbers of reviews and policymakers proposing each research question, as illustrated in the Venn diagram in figure 2. The diagram shows overlaps between the top 10 highest ranked questions from the Co-Digital exercise and the 10 most frequently mentioned research questions from the review articles and policymakers, respectively. Of the 30 questions, 21 were in the top 10 for at least one of the sources; 9 questions were in the top 10 for at least two sources. Two of the 30 questions were among the top 10 highest ranked for all three sources, including the question about effective strategies/mechanisms for implementing MSCs and the question about effective leadership, partnership and governance structures for MSCs.

10.1136/bmjgh-2018-000970.supp7 Supplementary data

Some questions were identified predominantly or exclusively through the reviews, such as the one related to study designs and methods, which was mentioned in 18 review articles, was not mentioned at all by the policymakers and ended up in the ninth slot in the online ranking. Other questions were more commonly suggested by policymakers, such as the question about how to increase MSC partner commitment, which was mentioned by eight policymakers and two review articles and did not make the top 10 ranking. Also of note, several of the questions that ranked highly in the online voting were only rarely mentioned in the reviews and by policymakers, such as the role of community-based partnerships and key challenges associated with implementing MSCs, each of which was only identified in one review and by one policymaker.

## Discussion

The objective of this paper was to provide guidance around research priorities to better understand MSCs and thus leverage them to improve population health and well-being, particularly in LMICs. In addition to yielding lists of priority MSC research topics identified in reviews, by policymakers and through a global, online ranking exercise, this study generated several other important insights worthy of reflection.

### Interest in MSCs spans a wide range of topical domains and disciplines

Nearly half of the reviews and a majority of policymakers discussed MSC from the perspective of a particular topical domain or issue, such as non-communicable diseases, food security and nutrition, HIV/AIDS, climate change or others. This underscores the instrumental nature of MSCs for achieving a variety of different health and social objectives and, by extension, the wide diversity of professional backgrounds and disciplines represented among those whose work has led them to develop an interest in MSC. Given this multiplicity of perspectives, the lack of apparent consensus around how best to conceptualise, implement and evaluate MSC is not surprising.

### Most research to date on MSCs focuses on HICs

The bulk of the MSC research from the literature search period (2000–2017)—most of which were published within the past 5–10 years—focused on HICs. Case in point, among the reviews that focused on the structure, process and/or mechanisms of MSC without respect to a particular topical area or domain, all of them either had a global focus, HIC focus, or a mixture of HIC and LMICs. None of these ‘cross-cutting’ articles had an exclusive LMIC focus. At the same time, there was a clear demand for MSC research from LMIC policymakers, many of whom mentioned MSC unprompted in response to an open-ended question about evidence needs for the SDGs during the consultations. While policymakers and practitioners from LMICs who are interested in MSCs will likely find some relevant material in the existing frameworks and guidance that draw primarily from HICs, there are also likely to be some significant contextual differences. For example, LMICs may have weaker public institutions, more limited resources, and a less clear delineation of roles and responsibilities, which may all undermine effective MSCs. At the same time, some of these differences may give rise to novel approaches to MSCs that may in some ways be more pragmatic, innovative and resourceful than similar efforts in HICs. In light of these likely institutional differences, there is an urgent need to take advantage of opportunities for MSC research in LMIC contexts.

### Fundamental questions about MSCs remain unanswered

The wide variation within the top 10 ranked questions combined with the relatively small spread in percentage scores between them suggests that there are many MSC questions worthy of attention and no strong consensus on which ones are the most pressing. This result might also suggest that there is quite a lot about MSCs that is not known or well understood—a hypothesis that is supported by the nature of many of the questions themselves, which, when distilled and summarised, might be paraphrased as ‘How do we initiate, structure, implement, sustain, and measure MSCs – and how do they affect health and social outcomes?’ These questions seem consistent with what one would expect in the early stages of a new research field, and yet we know that MSC has been an ongoing theme in efforts to improve the health of populations for over a century. One possible explanation for this discrepancy is that the framing, approaches, terminology and accumulated knowledge about MSCs are dispersed across numerous different epistemic communities and have only recently begun to coalesce—perhaps, at least in part, due to unifying global initiatives like UHC and frameworks like the SDGs.

### There is strong demand for practical, evidence-based guidance

The highly pragmatic focus of many of the questions—including the two questions that made it into the top 10 among the reviews, policymaker discussions and online voting—reinforces the earlier observation about the widespread framing of MSCs as a means to an end, and suggests that many of those asking these questions are seeking tangible, concrete and actionable guidance. This makes it all the more important for findings related to these priority research questions to be communicated in plain language that is accessible to non-researchers and across professional disciplines. Indeed, efforts to move forward the MSC research agenda may benefit substantially from close involvement of policymakers and practitioners, both in terms of shaping the specific research questions as well as interpreting and applying the results. Opportunities to do so may include country-level, multistakeholder knowledge platforms,^23–25^ embedded research,^26–29^ and expanded opportunities for applied, practice-based research training for policymakers,^30^ among others. The broad recognition of the context specificity of MSC (as evidenced by the seventh-ranked question ‘How do contextual factors…affect MSCs?’) points to a likely need for ongoing formative research as well as practical monitoring and evaluation frameworks and metrics to help shape and manage MSCs.

### Researchers want to know how best to study MSCs

A substantial gap in research methods for studying MSCs was apparent from both the expressed research needs (question 1 from the reviews and question 9 from the online ranking) as well as the fact that few of the review articles mentioned either the methodology of the resources/studies they reviewed or their own methodology for reviewing them. The apparent lack of agreement about the types of research approaches or methods that are appropriate to this space makes it difficult to answer other questions—such as those about what works, in what contexts, conditions, for what purpose and so on—and suggests that some foundational methodological work may be needed before some of the other priority questions can be answered. Synthesis and clarification of a common definition of MSC, key conceptual frameworks and a general typology of MSC structures may constitute useful steps in this direction.

Several strengths and limitations of this analysis are worth noting. One key strength was the ability to obtain input from a diverse and geographically dispersed set of researchers and policymakers around the globe. This was facilitated in part by the online platform for reviewing, editing and prioritising questions, which allowed respondents to contribute at their convenience and enabled greater overall participation than likely would have been financially and logistically feasible if the prioritisation exercise had been conducted in a face-to-face format. A related strength is the presentation of top suggestions for research priorities from each of the three phases (ie, reviews, policymaker consultations and online voting), which provides some insight into how the identification of priority research questions can vary depending on whose perspective is taken into account and the chosen methodology for prioritising. It also informs the reader of research questions that were frequently mentioned by either the reviews or policymakers but were not highly ranked by the health systems researchers who participated in the online voting and thus might otherwise be overlooked.

One notable limitation is the relatively low response rate from among those who were invited to reflect on the process and contribute to the ranking of research priorities. Another potential limitation is that policymakers were not asked directly about research priorities but rather about their policy priorities and associated evidence needs. These expressed evidence needs were then paraphrased in the form of research questions by the study team, which introduced an element of subjectivity. This decision was based on observations by other authors about the difficulty of eliciting research priorities directly from policymakers, who may feel more comfortable expressing information needs in the form of policy concerns rather than formal research questions.^11^ While this challenge could also have potentially been addressed through a deliberative, iterative discussion and refinement process,^17 18^ that was unfortunately not feasible given the time and resource constraints of this study. Similarly, the extraction of research questions from the reviews introduced a certain degree of subjectivity in the phrasing of the questions included in the prioritisation exercise. The limited representation from non-health sectors and junior-level policymakers, as well as the decision not to include other types of research users or beneficiaries (eg, health workers, community leaders and members, and so on) or to pursue a deliberative engagement process, should also be taken into account when interpreting the findings. Indeed, as we have noted above, there were notable variations in research priorities even between the three different sources included in this relatively focused study.

Given that this exercise was intended to provide a global picture of MSC research priorities—although with an emphasis on LMICs—it is unlikely to closely reflect the specific priorities of any particular country. Furthermore, the close competition between research questions for the top rankings indicates that there was no strong consensus on which ones should be tackled first. Both of these points underscore the need for local HRPS, which appears to be limited or absent in many LMICs around the world. This is illustrated by a 2014 systematic review by McGregor *et al*,^31^ which notes that although there has been a steady increase in documented reports of HRPS initiatives in LMICs since 2004, there remain substantial gaps. Case in point, the authors note that nearly half (46%) of the 91 LMIC-focused HRPS activities they found between 1999 and 2014 took place at a global level, and only 30 LMICs had done research priority-setting at the national level over the 15-year period.^31^ Additionally, just over half of the HRPS activities reviewed actually prioritised—as opposed to listing—research needs, and less than a quarter resulted in specific research questions.^31^

## Conclusions

Researchers, policymakers and practitioners from a wide range of disciplines have expressed a common view that MSCs serve a critical function in achieving a variety of health and social outcomes, yet the ways they describe, conceptualise and implement MSCs differ dramatically. Although MSC as a concept has been discussed in the global health community for over a century, the global push for UHC and the adoption of the SDGs have raised the profile of MSC in policy conversations. Our understanding of MSCs is still at an early stage of maturity globally (or at least that understanding has not coalesced into a consolidated body of knowledge), particularly in LMICs, and a variety of fundamental questions about MSCs—including how to initiate, structure, implement, sustain and measure them—remain unanswered. This shortage of empirical research and evidence-based guidance on MSCs, combined with the current window of opportunity afforded by the relatively high level of global interest in the topic, underscores the importance and timeliness of advancing this body of knowledge. This effort should draw on evidence from multiple professional disciplines, within and beyond the health sector, in both HICs and LMICs, and emphasise the bridging of research, policy and practice. Establishing a shared understanding of what MSC for health is and how to study it may be a prerequisite for addressing other identified MSC research priorities.
