# Supplementary material for: Identifying health policy and systems research priorities on multisectoral collaboration for health in low-income and middle-income countries
Source: BMJ Glob Health. 2018 Oct 10;3(Suppl 4):e000970. doi: 10.1136/bmjgh-2018-000970 (PMC6195136; doi:10.1136/bmjgh-2018-000970)
Supplement: Supplementary data [file bmjgh-2018-000970supp001.pdf]

Appendix 1: Search terms for literature review

Years: 2000-2017

Concept 1a:
("Intersectoral Collaboration"[Mesh] OR multisector*[tiab] OR "multi sector"[tiab] OR “inter sectoral”[tiab] OR “inter sector”[tiab] OR intersector*[tiab] OR inter-sector*[tiab] OR cross-sector*[tiab] “cross sectors”[tiab] OR “cross sectoral”[tiab] OR "cross-boundary"[tiab] OR “cross-boundaries”[tiab] OR collaboration[tiab] OR collaborations[tiab] OR integration[tiab] OR harmonious[tiab])

AND

Concept 1b:
((integration[tiab] OR collaboration[tiab] OR coordinate[tiab]) AND (sectoral[tiab] OR sector[tiab])) OR governance[tiab] OR convergence[tiab] OR cooperation[tiab] OR partnership[tiab] OR partnerships[tiab] OR policies[tiab] OR "Policy"[Mesh] OR policy[tiab] OR approach[tiab] OR program[tiab] OR programs[tiab] OR programme[tiab] OR programmes[tiab] OR strategic[tiab] OR implement*[tiab] OR goals[tiab] OR objective[tiab] OR objectives[tiab] OR outcome[tiab] OR outcomes[tiab] OR issue[tiab] OR issues[tiab]

AND

Concept 2:
(health[tiab] OR disease[tiab] OR nutrition[tiab] OR "undernutrition"[tiab] OR "malnutrition"[tiab] OR healthcare[tiab] OR "Public Health"[Mesh])

AND

Concept 3:
(government[tiab] OR "governance"[tiab] OR public[tiab] OR nation[tiab] OR "national"[tiab] OR "multinational"[tiab] OR international[tiab] OR “population”[tiab])

AND

Concept 4:
"review"[tiab] OR "meta-analysis"[tiab] OR "metaanalysis"[tiab] OR "systematic literature"[tiab] OR "meta synthesis"[tiab] OR "metasynthesis"[tiab]
