# Supplementary material for: Identifying health policy and systems research priorities on multisectoral collaboration for health in low-income and middle-income countries
Source: BMJ Glob Health. 2018 Oct 10;3(Suppl 4):e000970. doi: 10.1136/bmjgh-2018-000970 (PMC6195136; doi:10.1136/bmjgh-2018-000970)
Supplement: Supplementary data [file bmjgh-2018-000970supp002.pdf]

Appendix 2: Discussion of choice of search terms

The search strategy involved selecting articles in which the title or abstract matched a combination of controlled vocabulary and keyword terms such that at least one term was matched for all of the following concepts:

(1) inter-/multi-sectoral
(2) health or nutrition
(3) government/public
(4) review/synthesis

While the first and fourth search concepts are self-explanatory, the second and third warrant some additional explanation. For the second search concept, nutrition was added alongside health because of our observation that multiple nutrition-focused review articles deemed directly relevant to MSC for health did not explicitly include the word "health", and thus would have been excluded from the literature search results. The third search concept reflects our interest in identifying articles most relevant to building "country capacity", a term which we operationalize as the ability of one or more country's health system stakeholders, including (but not limited to) the government, to effectively lead or participate in a structured multi-sectoral effort (i.e., involving the health sector and at least one non-health sector) to improve the health and/or well-being of residents within the country's borders. Recognizing that a given country's health system is comprised of myriad stakeholders, both within and beyond the public sector, we take the position that the government is an essential actor both for sustainable MSC as well as achievement of the SDGs. This may include national-level governmental authorities, such as a ministry of health, but could also include governmental authorities at lower administrative levels, such as a state or provincial health office, provided that they have sufficient autonomy to set and enforce policy related to the collaboration. By this reasoning, MSCs not meeting these criteria such as donor-funded, NGO-implemented projects without substantive government involvement, were not included.
