# Supplementary material for: Identifying health policy and systems research priorities on multisectoral collaboration for health in low-income and middle-income countries
Source: BMJ Glob Health. 2018 Oct 10;3(Suppl 4):e000970. doi: 10.1136/bmjgh-2018-000970 (PMC6195136; doi:10.1136/bmjgh-2018-000970)
Supplement: Supplementary data [file bmjgh-2018-000970supp003.pdf]

Appendix 3: Data fields extracted for literature review

We used a Microsoft Office Excel based template to abstract key information from articles (including those directly and indirectly focused on multi-sectoral collaboration), including the following:

- Author(s) (automatically extracted)
- Title (automatically extracted)
- Date (automatically extracted)
- Journal (automatically extracted)
- Language (i.e., English, Spanish, or Portuguese)
- Institutional affiliation of first author
- Institutional affiliation of last author
- Review type (e.g., systematic review, review, not a review, etc.)
- Core research/review question (e.g., what processes support and inhibit multi-sectoral collaboration? what is the nature and extent or multi-sectoral coordination needed vs. in place for a particular intervention?)
- Definition of multi/intersectoral collaboration, if provided (and author/reference)
- Health-related objective/outcome(s) of interest (e.g., malaria, nutrition, health promotion, health equity, etc. - many of the reviews seem to be grouped in this way, but if the constituent papers address different topics, we could potentially either list them, or say "multiple")
- Key actors/sectors involved (e.g., health, agriculture, environment, etc.; if the article is more explicit, we could include those details as well - e.g., National Ministry of Health, provincial government authority, national labor union, etc. - there seems to be a mix in terms of whether the reviews focus primarily on the sectors vs. actors within those sectors)
- Barriers to multi-sectoral collaboration for health
- Author reflection on implications for policy and practice
- Author reflection on research implications & priorities (related to multi-sectoral collaboration);

For the subset of articles classified as directly focused on multi-sectoral collaboration, we extracted additional information, including:

- Number of papers
- Number of papers from LMICs
- Country(ies) included (i.e., including all papers referenced in the review)
- Conceptual/theoretical framework, if applicable (e.g., Bergen Model of Collaborative Functioning)
- Targeted beneficiary group(s) (or "not stated")
- Administrative level(s) involved (e.g., international, national, state/regional, local)
- Methods in primary papers for assessing success/effectiveness of multi-sectoral collaboration for health
- Success factors for multi-sectoral collaboration for health
- Author assessment of quality of included papers
- Data extractor reflections on quality of review (e.g., clear search criteria; methods clearly defined; tracing of how papers were included and excluded)
