# Supplementary material for: Identifying health policy and systems research priorities on multisectoral collaboration for health in low-income and middle-income countries
Source: BMJ Glob Health. 2018 Oct 10;3(Suppl 4):e000970. doi: 10.1136/bmjgh-2018-000970 (PMC6195136; doi:10.1136/bmjgh-2018-000970)
Supplement: Supplementary data [file bmjgh-2018-000970supp004.pdf]

Appendix 4: Themes, research needs and research questions

The contents of the table are sorted first by Theme, then Research needs, then Research questions, according to the combined frequency of mention in the overview of reviews and policymaker consultations.

Theme	Research needs	Research questions
Stakeholders<br>*(40 mentions)*	Discussion/guidance on the key stakeholders (including public sector, private sector, community, etc.) and their respective roles/functions and relationships in multi-sectoral collaboration/policy to address various health issues in different countries/contexts (e.g., climate change, HIV/AIDS)<br>*(19 mentions)*	What is the appropriate role for the Ministry of Health in multi-sectoral collaborations vis-à-vis other ministries and how does this vary across topics/contexts?<br>*Mentioned by: 3 articles; 6 policymakers*
		How can initiators of multi-sectoral collaboration determine the appropriate scope of the partnership (e.g., number of partners to include, level of involvement of each)?<br>*Mentioned by: 1 article; 3 policymakers*
		What is the role of non-health decision makers in shaping the public health policy and research agenda?<br>*Mentioned by: 3 articles; 0 policymakers*
		What is the role of community-based partnerships and initiatives in driving multi-sectoral collaborations for health?<br>*Mentioned by: 1 article; 1 policymaker*
		What role can policy champions play in driving multi-sectoral collaborations and how can this be supported/scaled up?<br>*Mentioned by 1 article; 0 policymakers*
	Better understanding of how to generate collective ownership, buy-in, and political commitment across multiple actors/sectors<br>*(10 mentions)*	In formal multisector partnerships, what can be done to increase the commitment of members through incentives and other means?<br>*Mentioned by 2 articles; 8 policymakers*
	Guidance on multi-sectoral collaboration including both government and private sector stakeholders (e.g., public-private partnerships)<br>*(8 mentions)*	What are the main differences in multi-sectoral collaborations involving private sector partners vs. public sector only?<br>*Mentioned by 1 article; 7 policymakers*
	Understanding beneficiary/recipient/population experiences of multi-sectoral programs or initiatives<br>*(3 mentions)*	How do clients or beneficiaries experience effective (or less effective) multi-sectoral initiatives?<br>*Mentioned by 2 articles; 0 policymakers*
		How do multi-sectoral initiatives affect vulnerable and marginalized populations?<br>*Mentioned by 1 article; 0 policymakers*
Strategies/<br>Approaches/<br>Interventions<br>*(30 mentions)*	Identification of the most promising/appropriate/effective strategies, approaches or interventions for improving or expanding multi-sectoral collaboration<br>*(25 mentions)*	Which strategies and mechanisms are effective in supporting the implementation of multi-sectoral collaborations for health? (e.g., enabling legislation, policy mandate, decentralized control, accountability and incentive mechanisms, dedicated resources, training/skill development, etc.)<br>*Mentioned by 9 articles; 8 policymakers*
		How does multi-sectoral collaboration at the local level differ (e.g., in terms of challenges, processes) from the national level?<br>*Mentioned by: 1 article; 3 policymakers*

Theme	Research needs	Research questions
		Is there a set of core activities that facilitate multi-sectoral collaborations across contexts?<br>*Mentioned by: 2 articles: 0 policymakers*
		How can you take multi-sectoral collaboration from local level initiatives to scale?<br>*Mentioned by: 0 articles; 2 policymakers*
	Identification of the most promising/appropriate/effective strategies, approaches or interventions (e.g., "best practices") for addressing broader issues of health equity and social determinants in different countries/contexts<br>*(5 mentions)*	How can multi-sectoral collaborations improve health equity and social determinants of health?<br>*Mentioned by: 5 articles; 0 policymakers*
Structure/ Process/ Mechanism<br>*(23 mentions)*	Guidance on appropriate leadership and governance structures for initiating/guiding/managing multi-sectoral collaborations, including "how to" process details<br>*(23 mentions)*	What types of leadership, partnership and governance structures and processes are most effective for multi-sectoral collaboration?<br>*Mentioned by: 10 articles; 13 policymakers*
Measurement/ evaluation/ research methods<br>*(18 mentions)*	Guidance on which types of research frameworks, methods, data, data collection techniques, and/or partnerships are best suited for answering various research questions related to multi-sectoral collaborations, their governance structures, functioning, and outcomes<br>*(18 mentions)*	Which study designs and methods are best suited to understanding multi-sectoral collaborations, their governance, functioning and outcomes?<br>*Mentioned by: 18 articles; 0 policymakers*
Conditions/ Context<br>*(18 mentions)*	Identify contextual factors that affect the success of multi-sectoral collaboration (e.g., focused on climate change, Health in All Policies, nutrition, early childhood development) and their strategies/interventions in different places, at different levels (e.g., national vs. local), and over time<br>*(11 mentions)*	How do contextual factors such as institutional arrangements, governance arrangements, partnership experiences affect the success (or failure) of multi-sectoral collaborations?<br>*Mentioned by: 10 articles; 1 policymaker*
	Identify necessary conditions or circumstances (if any) to form or initiate multi-sectoral collaborations<br>*(6 mentions)*	What are they key conditions or drivers for the formation of multi-sectoral partnerships (e.g., political context, motivating factors for partners, etc.)?<br>*Mentioned by 6 articles; 0 policymakers*
	Identify criteria for determining when multi-sectoral collaboration is necessary and/or useful<br>*(1 mention)*	Under what conditions are multi-sector partnerships necessary or more effective than other strategies for population health improvement?<br>*Mentioned by: 1 article; 0 policymakers*
Effectiveness/ Impact<br>*(13 mentions)*	Assessment of the progress, effectiveness, and/or impact of specific multi-sectoral interventions to address various health or health systems issues in different countries/contexts<br>*(11 mentions)*	What is the additional impact of multi-sectoral collaboration on health and health equity outcomes as compared to single sector approaches?<br>*Mentioned by: 8 articles; 3 policymakers*
	Identify common or potential unintended consequences (positive or negative) from multi-sectoral initiatives<br>*(2 mentions)*	What are the unintended consequences (positive or negative) of multi-sectoral partnerships?<br>*Mentioned by: 2 articles; 0 policymakers*
Inputs/Capacity/ Resources<br>*(9 mentions)*	Identification and description of the capacity needs (e.g., individual, organizational) associated with various multi-sectoral strategies or interventions<br>*(7 mentions)*	How can we best enhance the capacity of stakeholders concerned about multi-sectoral action for health (such as health advocates, or health practitioners), to engage in multi-sectoral initiatives?<br>*Mentioned by: 5 articles; 2 policymakers*

Theme	Research needs	Research questions
	Identification and description of the required inputs (e.g., human, financial, material) for various multi-sectoral strategies or interventions<br>*(2 mentions)*	What are the resource costs of initiating and maintaining multi-sectoral collaborations (e.g., as opposed to single-sector interventions)?<br>*Mentioned by: 1 article; 1 policymaker*
Inter-sectoral effects<br>*(8 mentions)*	Description and quantification of how health affects other sectors (e.g., education, labor, others) and vice versa<br>*(8 mentions)*	What is the impact of good health or health services on the ability of other sectors (outside of health) to achieve their Sustainable Development Goals?<br>*Mentioned by: 1 article; 3 policymakers*
		How do/will interventions targeting non-health SDGs affect health outcomes?<br>*Mentioned by: 3 articles; 1 policymaker*
Data sharing<br>*(8 mentions)*	Guidance on harmonizing research and monitoring and evaluation of multi-sectoral issues and/or initiatives across multiple sectors and professional fields<br>*(8 mentions)*	How can indicators and information systems be harmonized across partners in a multi-sectoral collaboration?<br>*Mentioned by: 3 articles; 5 policymakers*
Sustainability<br>*(5 mentions)*	Description/analysis/guidance on sustaining multi-sectoral collaborations over time<br>*(5 mentions)*	What are the factors that help to sustain multi-sectoral collaborations over time?<br>*Mentioned by: 1 article; 4 policymakers*
Framework/ Theory/ Definitions<br>*(4 mentions)*	Discussion/consideration of the most useful or appropriate framework(s) or theory(ies) for understanding and/or measuring multi-sectoral issues and interventions<br>*(4 mentions)*	Which theories and/or conceptual frameworks are most valuable in understanding multi-sectoral issues?<br>*Mentioned by: 4 articles; 0 policymakers*
Role of evidence<br>*(4 mentions)*	Identification and description of different evidentiary standards across sectors and how that affects collaboration<br>*(4 mentions)*	How does the use of evidence differ across different sectors and how can we make health evidence more accessible and actionable in other sectors?<br>*Mentioned by: 4 articles; 0 policymakers*
Challenges/ Obstacles<br>(2 mentions)	Identify key challenges/barriers to implementing various components of multi-sectoral packages to address health issues (e.g., food security, NCDs, HIV/AIDS) and/or health equity<br>*(2 mentions)*	What are the key barriers to implementing multi-sectoral programs to address health issues (e.g., food security, NCDs, HIV/AIDS)?<br>*Mentioned by: 1 article, 1 policymaker*
