# Supplementary material for: Identifying health policy and systems research priorities on multisectoral collaboration for health in low-income and middle-income countries
Source: BMJ Glob Health. 2018 Oct 10;3(Suppl 4):e000970. doi: 10.1136/bmjgh-2018-000970 (PMC6195136; doi:10.1136/bmjgh-2018-000970)
Supplement: Supplementary data [file bmjgh-2018-000970supp005.pdf]

Appendix 5: Policymaker consultation respondents

Policymaker respondents represented a variety of different types of institutions, including: state government; office of the prime minister; ministry of planning; ministry of health; department of health; department of environmental affairs; department of public service and administration; national council for coordination of social policies; public health agencies; various bilateral and multilateral development organizations; various private or not-for-profit technical implementers of development aid; and others.

Positions included: minister for health; principal secretary for health; health commissioner and assistant commissioner; director-general and deputy director-general for health; directors of various departments; secretaries and assistant secretaries; special advisors; senior planning officers; senior policy analysts; organizational presidents and vice presidents; technical leads; project coordinators; and others.

Of the 54 respondents included in IDIs, 15 (28%) were female and 39 (72%) were male. Details on the gender of focus group participants were not recorded.

IDI respondent summary by geographic region

WHO Region	Total No. Respondents Identified & Invited	Total No. Respondents Included	Total No. Respondents Discussing MSC	Interview Language(s)
Africa Region	30	12	10	English
Region of the Americas	11	10	10	English, Spanish
South-East Region	17	14	14	English
European Region	0	0	0	NA
Eastern Mediterranean Region	5	4	3	English, Arabic, French
Western Pacific Region	15	8	7	English, Mandarin
Multi/Bi-lateral Org/NGOs	7	6	4	English
**Total**	**85**	**54**	**48**	

FGD respondent summary

Focus Group Country	No. of Respondents Invited	Total No. Respondents Included	Focus Group Discussion Language
Bahrain	16	10	English
Jordan	17	17	Arabic
**Total**	**33**	**27**	
