# Supplementary material for: Identifying health policy and systems research priorities on multisectoral collaboration for health in low-income and middle-income countries
Source: BMJ Glob Health. 2018 Oct 10;3(Suppl 4):e000970. doi: 10.1136/bmjgh-2018-000970 (PMC6195136; doi:10.1136/bmjgh-2018-000970)
Supplement: Supplementary data [file bmjgh-2018-000970supp006.pdf]

Appendix 6: Top thirty ranked research questions on multi-sectoral collaboration for health

Rank	Research Question	Unweighted final score
1	Which strategies and mechanisms are effective in supporting the implementation of multisectoral collaborations for health? (e.g., enabling legislation, policy mandate, decentralized control, accountability and incentive mechanisms, dedicated resources, training/skill development, etc.)	67.9%
2	What factors are necessary for sustaining multisectoral collaborations over time?	63.0%
3	How does the use of evidence differ across different sectors and how can we make health evidence more accessible and actionable in other sectors?	62.7%
3	What is the role of community-based partnerships and initiatives in driving multi-sectoral collaborations for health?	62.7%
5	What types of leadership, partnership, and governance structures and processes are most effective for multisectoral collaboration?	60.0%
6	What are the key challenges to implementing multisectoral programs and interventions to address health issues (e.g., food security, NCDs, HIV/AIDS)?	59.7%
7	How do contextual factors such as institutional arrangements, governance arrangements, democratic values, partnership experiences affect the success (or failure) of multisectoral collaborations?	53.0%
8	How can we best improve the capacity of stakeholders involved in multisectoral action for health (such as health advocates, or health practitioners), to engage in and also promote multisectoral initiatives?	52.6%
9	Which study designs and methods are best suited to understanding multisectoral collaborations, their governance, functioning and outcomes?	51.8%
10	How do multisectoral collaborations affect health equity and social determinants of health?	50.9%
10	How do interventions that target non-health SDGs affect health outcomes?	50.9%
12	What are the drivers that bring actors together to form multi-sectoral partnerships (e.g., political context, organizational objectives, other motivating factors, etc.)?	49.2%
13	For which health issues are multi-sectoral partnerships more effective than other strategies?	48.5%
14	What is the role of non-health decision makers in shaping the public health policy and research agenda?	48.3%
15	How can indicators and information systems be harmonized across partners in a multisectoral collaboration?	46.0%
16	What is the impact of good health or health services on the ability of other sectors (outside of health) to achieve their Sustainable Development Goals?	45.1%
17	How do multisectoral initiatives affect vulnerable and marginalized populations?	44.8%
18	Which conceptual and theoretical frameworks can further contribute to understanding multisectoral issues?	44.4%
19	What role can policy champions play in driving multi-sectoral collaborations and how can this engagement be supported?	42.9%
20	What is the additional impact of multisectoral collaboration on health and health equity outcomes as compared to single sector approaches?	40.0%
21	What are the resource costs of initiating and maintaining multi-sectoral collaborations (e.g., as opposed to single-sector interventions)?	39.6%
22	What can be done in formal multi-sector partnerships to increase the commitment of members through incentives and other means?	38.9%
23	How can initiators of multi-sectoral collaborations determine the appropriate scope of the partnership (e.g., number of partners to include, level of involvement of each)?	38.5%
24	How does multisectoral collaboration differ from the local to the national level (e.g., in terms of challenges, processes)?	36.4%
25	What are the main differences between multi-sectoral collaborations involving private sector partners or multi-sectoral collaborations involving public sector only?	33.3%

Rank	Research Question	Unweighted final score
25	Is there a set of core activities that facilitate multi-sectoral collaborations across contexts, and if so, what are they?	33.3%
27	What is the role of the Ministry of Health in multi-sectoral collaborations vis-Ã -vis other ministries and how does this vary across topics/contexts?	27.3%
28	How do clients or beneficiaries experience effective (or less effective) multisectoral initiatives?	25.0%
29	What are the unintended consequences (positive or negative) of multi-sectoral partnerships?	20.0%
30	How can locally initiated multisectoral collaborations be scaled up?	18.2%
