# Supplementary material for: Identifying health policy and systems research priorities on multisectoral collaboration for health in low-income and middle-income countries
Source: BMJ Glob Health. 2018 Oct 10;3(Suppl 4):e000970. doi: 10.1136/bmjgh-2018-000970 (PMC6195136; doi:10.1136/bmjgh-2018-000970)
Supplement: Supplementary data [file bmjgh-2018-000970supp007.pdf]

Appendix 7: Full list of priority research questions from reviews and policymaker consultations

Questions in the table are sorted according to the combined frequency of mention in the overview of reviews and policymaker consultations.

Final question	Original question	# generations	# of mentions in reviews	# of mentions in policymaker consultations
What types of leadership, partnership, and governance structures and processes are most effective for multi-sectoral collaboration?	What types of leadership, partnership and governance structures and processes are most effective for multi-sectoral collaboration?	3	5: (23,27,46,49,55)	12: Caribbean, S. Africa, India, Indonesia, IADB, Somalia, PAHO, World Bank, India, Myanmar
Which strategies and mechanisms are effective in supporting the implementation of multi-sectoral collaborations for health? (e.g., enabling legislation, policy mandate, decentralized control, accountability and incentive mechanisms, dedicated resources, training/skill development, etc.)	Which strategies and mechanisms are effective in supporting the implementation of multi-sectoral collaborations for health? (e.g., enabling legislation, policy mandate, decentralized control, accountability and incentive mechanisms, dedicated resources, training/skill development, etc.)	2	7: (28,29,35,39,40,47,54)	8: Argentina, India, Caribbean, Thailand, WHO, India, Jordan, South Africa
Which study designs and methods are best suited to understanding multi-sectoral collaborations, their governance, functioning and outcomes?	Which study designs and methods are best suited to understanding multi-sectoral collaborations, their governance, functioning and outcomes?	1	11: (23,29,30,33,42,44,45,51,53,54,56)	
How do contextual factors such as institutional arrangements, governance arrangements, democratic values, partnership experiences affect the success (or failure) of multi-sectoral collaborations?	How do contextual factors such as institutional arrangements, governance arrangements, partnership experiences affect the success (or failure) of multi-sectoral collaborations?	3	9: (23,27,32,35,38,44–46,54)	1: India
What is the role of the Ministry of Health in multi-sectoral collaborations vis-à-vis other ministries and how does this vary across topics/contexts?	What is the appropriate role for the Ministry of Health in multi-sectoral collaborations vis-à-vis other ministries and how does this vary across topics/contexts?	2	3: (41,44,48)	6: Indonesia, Bahrain, Argentina, World Bank, Myanmar, China
What is the additional impact of multi-sectoral collaboration on health and health equity outcomes as compared to single sector approaches?	What is the additional impact of multi-sectoral collaboration on health and health equity outcomes as compared to single sector approaches?	3	7: (23,25,36,41,43,45,49)	2: India, Argentina
How can indicators and information systems be harmonized across partners in a multi-sectoral collaboration?	How can indicators and information systems be harmonized across partners in a multi-sectoral collaboration?	1	3: (52–54)	5: Bhutan, India, Kiribati, Argentina
What can be done in formal multi-sector partnerships to increase the commitment of members through incentives and other means?	In formal multisector partnerships, what can be done to increase the commitment of members through incentives and other means?	4	2: (23,54)	6: Bhutan, South Africa, Kenya, Caribbean, Laos, Thailand

Final question	Original question	# gene-rations	# of mentions in reviews	# of mentions in policymaker consultations
How can we best improve the capacity of stakeholders involved in multi-sectoral action for health (such as health advocates, or health practitioners), to engage in and also promote multi-sectoral initiatives?	How can we best enhance the capacity of stakeholders concerned about multi-sectoral action for health (such as health advocates, or health practitioners), to engage in multi-sectoral initiatives?	3	5: (27,28,35,51,57)	2: Bahrain, South Africa
What are the main differences between multi-sectoral collaborations involving private sector partners or multi-sectoral collaborations involving public sector only?	What are the main differences in multi-sectoral collaborations involving private sector partners vs. public sector only?	3	1: (25)	5: Liberia, Jordan, India, Argentina
What is the impact of good health or health services on the ability of other sectors (outside of health) to achieve their Sustainable Development Goals?	What is the impact of good health or health services on the ability of other sectors (outside of health) to achieve their Sustainable Development Goals?	3	1: (31)	4: Pakistan, Laos, Thailand, PAHO
What factors are necessary for sustaining multi-sectoral collaborations over time?	What are the factors that help to sustain multi-sectoral collaborations over time?	5	1: (23)	4: Indonesia, Kiribati, IADB, Somalia
What are the drivers that bring actors together to form multi-sectoral partnerships (e.g., political context, organizational objectives, other motivating factors, etc.)?	What are the key conditions or drivers for the formation of multi-sectoral partnerships (e.g., political context, motivating factors for partners, etc.)?	4	4: (23,27,51,54)	
Which conceptual and theoretical frameworks can further contribute to understanding multi-sectoral issues?	Which theories and/or conceptual frameworks are most valuable in understanding multi-sectoral issues?	5	4: (26,27,49,55)	
How do interventions that target non-health SDGs affect health outcomes?	How do/will interventions targeting non-health SDGs affect health outcomes?	4	2: (31,45)	1: Kenya
How does multi-sectoral collaboration differ from the local to the national level (e.g., in terms of challenges, processes)?	How does multi-sectoral collaboration at the local level differ (e.g., in terms of challenges, processes) from the national level?	2	1: (51)	2: IADB, India
How can initiators of multi-sectoral collaborations determine the appropriate scope of the partnership (e.g., number of partners to include, level of involvement of each)?	How can initiators of multi-sectoral collaboration determine the appropriate scope of the partnership (e.g., number of partners to include, level of involvement of each)?	3	1: (33)	2: Myanmar, Indonesia
What is the role of non-health decision makers in shaping the public health policy and research agenda?	What is the role of non-health decision makers in shaping the public health policy and research agenda?	1	3: (32,33,44)	0

**Final question**	**Original question**	**# gene-rations**	**# of mentions in reviews**	**# of mentions in policymaker consultations**
How does the use of evidence differ across different sectors and how can we make health evidence more accessible and actionable in other sectors?	How does the use of evidence differ across different sectors and how can we make health evidence more accessible and actionable in other sectors?	3	3: (32,34,55)	0
How do multi-sectoral collaborations affect health equity and social determinants of health?	How can multi-sectoral collaborations improve health equity and social determinants of health?	2	3: (28,54,57)	0
What are the key challenges to implementing multi-sectoral programs and interventions to address health issues (e.g., food security, NCDs, HIV/AIDS)?	What are the key barriers to implementing multi-sectoral programs to address health issues (e.g., food security, NCDs, HIV/AIDS)?	5	1: (54)	1: Caribbean
What are the resource costs of initiating and maintaining multi-sectoral collaborations (e.g., as opposed to single-sector interventions)?	What are the resource costs of initiating and maintaining multi-sectoral collaborations (e.g., as opposed to single-sector interventions)?	2	1: (36)	1: Bhutan
What is the role of community-based partnerships and initiatives in driving multi-sectoral collaborations for health?	What is the role of community-based partnerships and initiatives in driving multi-sectoral collaborations for health?	2	1: (36)	1: Ghana
How can locally initiated multi-sectoral collaborations be scaled up?	How can you take multi-sectoral collaboration from local level initiatives to scale?	4	0	2: Bhutan, IADB
What are the unintended consequences (positive or negative) of multi-sectoral partnerships?	What are the unintended consequences (positive or negative) of multi-sectoral partnerships?	1	2: (23,36)	0
Is there a set of core activities that facilitate multi-sectoral collaborations across contexts, and if so, what are they?	Is there a set of core activities that facilitate multi-sectoral collaborations across contexts?	3	2: (21,36)	0
For which health issues are multi-sectoral partnerships more effective than other strategies?	Under what conditions are multi-sector partnerships necessary or more effective than other strategies for population health improvement?	5	1: (23)	0
What role can policy champions play in driving multi-sectoral collaborations and how can this engagement be supported?	What role can policy champions play in driving multi-sectoral collaborations and how can this be supported/scaled up?	3	1: (54)	0
How do clients or beneficiaries experience effective (or less effective) multi-sectoral initiatives?	How do clients or beneficiaries experience effective (or less effective) multi-sectoral initiatives?	2	1: (39)	0
How do multi-sectoral initiatives affect vulnerable and marginalized populations?	How do multi-sectoral initiatives affect vulnerable and marginalized populations?	2	1: (43)	0
